# Flood vulnerability and economic valuation of small and medium-sized enterprise owners to enhance sustainability

**DOI:** 10.4102/jamba.v14i1.1306

**Published:** 2022-09-28

**Authors:** Muzakar Isa, Ahmad Mardalis

**Affiliations:** 1 Department of Management, Faculty of Economic and Business, Muhammadiyah University of Surakarta, Surakarta, Indonesia

**Keywords:** flood risk, willingness to pay, economic valuation, SME’s, vulnerability

## Abstract

Small and medium-sized enterprises (SMEs) play an important role in supporting Indonesia’s economic growth and provide employment for people. Nevertheless, SMEs are most vulnerable when there is a flood. Small and medium sized enterprises are worse off especially after the flood, because they are relatively limited in resources and less resilient. The study aimed at identifying the vulnerability level to floods and analysing the economic valuation of flood mitigation. The population in this study were SMEs located in flood-prone areas in Klaten Regency, Central Java province, Indonesia. This research used a purposive sampling technique with 152 respondents. Data collection was carried out using a direct interview method to business actors with the help of a questionnaire. There were two analytical tools used in this research, including vulnerability index and economic valuation. The results showed that Klaten Regency is located in the upper area vulnerability category of moderate flood. The economic valuation of flood mitigation is IDR 100 000 (USD6.99) to IDR 149 999 (USD10.49). Most SMEs perceive that flood mitigation is the responsibility of the government.

## Introduction

Small and medium enterprises (SMEs) have a vital role in supporting Indonesia’s economic growth, in which their number has reached 62.92 million units or approximately 99.99% of the total existing business actors. They contribute about 57.08% of GDP and provide around 117 million jobs for the people of Indonesia (BPS 2018). Nevertheless, they are the most affected units in the event of a flood. The shortage of risk analysis and the complexity of post-disaster recovery are some significant issues for them. Isa and Mangifera (2019) make it clear that SMEs are most vulnerable to financial difficulties because they often lack the resources to adapt when there is a flood. In developing countries, the impact of flooding can be worse. Small and medium enterprises fare worse especially after the flood because they are relatively limited in resources and less resilient (Isa & Mangifera 2019). They also have limited access to a broader set of coping strategies and are generally unprepared for natural hazards. Most SMEs do not have insurance and do not carry out risk assessments; they usually do not have a business continuity plan (Ye & Abe 2012). Most small businesses are also characterised by informality. Informality also impedes the ability of SMEs to expand their customer base and supply (Setyawan et al. 2018). It is essential that they pay particular attention to their finances (Leitold et al. 2021), especially regarding potentially serious problems that are not always immediately visible. Yet small firms may prove that they can successfully pass through this period, having certain advantages compared to the large ones.

Indonesia ranks sixth amongst the world’s most vulnerable countries to the risk of flooding (Isa 2016; Isa, Sugiyanto & Susilowati 2018). Based on the data from the National Agency for Disaster Countermeasure (BNPB) of 2019, Indonesia experienced 24 437 natural hazards during 1815–2018. Floods are the most common natural hazards, as indicated by 37.284% of the total natural hazards in Indonesia. They are followed by tornados at 25.94%, landslides at 21.07%, drought at 8.18%, fires at 3.95%, earthquakes at 1.40%, abrasion at 1.28%, volcanic eruptions at 0.79%, tsunamis at 0.08% and a combination of earthquake and tsunami at 0.01%. Furthermore, Central Java is the province with the highest vulnerability to floods, as indicated by the occurrence of 505 floods in the last 5 years. Meanwhile, Klaten is one of the regencies in Central Java that often experiences floods, although it is not located in the coastal area (BNPB 2019). In 2014–2018, there were 18 floods in Klaten regency. This hazard has brought much damage, in which approximately 4219 people were evacuated, 1059 buildings were inundated and 817 ha of rice paddy fields were affected (BNPB 2019).

Flood is a hazard with a large number of potential damages and losses. Nevertheless, in case there is a threat of flooding in a low vulnerability area, the SMEs tend to have the capacity to overcome it so that the risks can be minimised. Meanwhile, in a moderate or high vulnerability level area, the risk of flooding will be higher. It means that vulnerability is the main factor that causes the risk of flooding (Isa 2016; Isa, Sugiyanto & Susilowati 2015). Therefore, various efforts to reduce the risk of flooding through the reduction of the vulnerability index of an area or so-called flood mitigation are required.

Floods definitely have adverse impact on the short-run regional economic development; hence, mitigation activities must be promoted to reduce the impact (Isa 2016). Nevertheless, mitigation will be optimal once the people realise the significance of such activities and actively contribute towards the efforts at the same time. The consciousness level of the SMEs owner in the flood-prone areas is reflected by the willingness to pay (WTP) for them to support mitigation activities. An economic valuation to assess the benefits and effects generated from mitigation activities is vital for decision-making and economic analysis of an area. A number of economists have developed methods to estimate the economic value of environmental and natural resources, particularly for non-market goods and services (Kim 2002). This valuation can be done through various methods and techniques (Arnal et al. 2016). Economic valuation for mitigation of flood-prone areas may involve market and non-market valuation approaches. Market valuation approaches include productivity methods, human capital or lost production and the opportunity cost method. Non-market valuation methods include preference methods, such as the hedonic price method, travel cost method, WTP method and benefit transfer method (Susilowati et al. 2019).

An approach often employed in the economic valuation is the WTP or willingness to accept compensation (Arnal et al. 2016; Reynaud & Nguyen 2016). It is used to estimate the economic value of ecosystem and environmental services that have no market value (Botzen & Bergh 2012); for instance, scenic beauty. It uses the WTP or willingness to accept compensation method so that the natural resources are not harmed. It is included as a preference method because it allows people to express their assessment and appreciation (Rewitzer et al. 2017). It also reveals the magnitude of concern for environmental goods and services based on their benefits for all parties, so that conservation efforts are important to maintain the benefits (Islam, Kotani & Managi 2016).

Susilowati et al. (2019) and Rewitzer et al. (2017) carried out several activities in economic valuation. These include the following:

1. Preparing questionnaires for surveys on the benefits of natural resources.
2. Conducting a survey on specific respondents. In this survey, questions are processed into market variables, namely the WTP as expressed in money values and compensation that represents benefits if the natural resources and environmental services are lost.
3. Processing survey results as the derivation of the average demand curve of respondent for natural resources.
4. Estimating the average value per individual of SMEs, and extrapolating it with the population so that the total benefits of an environmental service can be determined.

The study aimed at identifying the vulnerability level to floods and analysing the economic valuation of flood mitigation.

## Flood risk and vulnerability

The high risk of flooding hurts the local income and labour absorption, and it ultimately has adverse effects on the regional economic growth (Isa et al. 2015). Regional economic growth is the primary indicator of the achievement of regional development. Isa et al. (2015) described that the frequency and duration of floods influence the magnitude of the risk of flooding. Inevitably, flood brings much damage to the factors of production and losses that positively affect economic growth. Flood risk reduction should be undertaken to maintain regional economic growth.

Swart and Frank (2007), Isa et al. (2018), and Isa et al. (2019) explicated that the risk of flooding is linked to hazards and vulnerability. Vulnerability is a condition that causes the incapability of SMEs in facing the peril of flood. It is assumed as a significant determinant of disaster risk, because a hazard does not necessarily bring any risk unless it interacts with the vulnerable physical, social and economic environment (McEntire 2012). The attempt to reduce flood risk on SMEs can be made by diminishing the vulnerability of a region. It requires the identification of regional vulnerability to the flood. Douben (2006), Smit and Wandel (2006), Akukwe and Ogbodo (2015) and Isa et al. (2018) suggested that flood area vulnerability consists of three aspects: exposure, sensitivity and adaptive capacity.

Vulnerability is understood as the tendency of an element to be negatively affected because of external causes, namely flooding. Vulnerability has a deep quantitative character and represents damage and loss of items (person, equipment, economic or social capital, etc.) exposed to certain risks after a flood. Vulnerability is expressed on a scale from 0 to 1. Vulnerability can be temporary or permanent, depending on the difficulty of the problem that must be addressed, namely internal and external organisation, according to the factors that determine vulnerability.

### Flood vulnerability and small and medium-sized enterprises

According to Auzzira, Haigh and Amaratunga (2018), the Bolton Committee in 1971 was the first organisation to provide the definition of SME. Small and medium enterprises were defined as follows:

[*A*] firm is regarded as small if it meets the following three criteria, such as, it has relatively small share of the market place, it is managed by owners in a personalized way management structure, and it doesn’t form part of a large enterprise. (p. 1131–1138)

Small and medium enterprises are defined differently from one country to another. Each definition of SMEs uses different criteria and indicators. This study uses a definition supported by the Indonesian Central Statistics Agency (BPS). BPS provides a definition of SMEs based on labour force. Small businesses are business entities that have a workforce of 5–19 people, whilst medium businesses are business entities that have a workforce of 20–99 people.

Many SMEs prefer to operate in urban areas because of high growth of urban markets (Chatterjee, Ismail & Shaw 2016). Urban areas in general are areas that are prone to flooding (Auzzira et al. 2018). Business operations of SMEs in flood-prone areas will provide higher vulnerability (Pathak & Ahmad 2016). Furthermore, mitigation and institutions are other factors that contribute greatly to increasing the vulnerability of SMEs to floods (Isa et al. 2015, 2021). Falkner and Hiebl (2015) and Auzzira et al. (2018) stated that SMEs were affected by flooding, mainly because of the lack of awareness of SME actors in business risk analysis. Neise and Revilla Diez (2019) stated that firms’ flood adaptation strategies differ regarding business size. The large and medium-sized firms can adapt more effectively to floods, whilst small firms face difficulties because of inferior dynamic capabilities. Hashim et al. (2021) report that risk perception and males are the most consistent factors in influencing flood preparedness actions.

Small and medium enterprises play an important role in supporting Indonesia’s economic growth. Small and medium enterprises account for over 99% of all enterprises. They contribute significantly to economic growth, with their share of GDP ranging 57% in Indonesia economies (BPS 2018). These SMEs also contribute a major share to the GDP (Pathak & Ahmad 2016) and are to be protected from the disastrous impacts of the recurring floods (Auzzira et al. 2018). During floods, SMEs are the most vulnerable business units, and many SMEs suffer losses (Pathak & Ahmad 2016). Even though SMEs often operate under difficult conditions, they are surprisingly often willing to contribute to flood risk reduction (Neise, Sambodo & Revilla Diez 2019).

## Research method

Klaten is a regency in the Central Java province of Indonesia, situated at the coordinates of 7˚32′19″–7˚41′33″S and 110˚26′14″–110˚47′51″E. Administratively, Klaten regency is divided into 26 subdistricts, 391 villages and 10 *kelurahan* [administrative villages]. Klaten regency is also divided into three plains, namely Mount Merapi slope, limestone mountain and lowland. Regencies in Central Java province are included as flood-prone areas, with the highest risks for the category of non-coastal areas. Based on the Klaten Regency Spatial Plan for 2011–2031, several subdistricts are included as flood-prone areas, namely Bayat, Cawas, Ceper, Gantiwarno, Juwiring, Karangdowo, Pedan, Prambanan, Trucuk, Wedi and Wonosari.

The population of the present study included the SMEs in the flood-prone areas of Klaten Regency, Central Java, Indonesia. Nevertheless, the precise number of the population is unidentified because of the unavailability of such information. This study used a purposive sampling technique involving 152 respondents. Direct survey techniques were carried out on SMEs in the manufacturing sector in flood-prone areas in Klaten Regency, namely rice mills, furniture, handicrafts, confections and *batik* [a technique of wax-resist dyeing applied to the whole cloth].

The present study used primary data that could be divided into the following categories: (1) the individual data of respondents, such as age, gender and educational background; (2) the characteristics of SMEs, namely type of business, legal entity, number of workers, distance to river, production costs and products; (3) the knowledge level of SMEs about floods and their risks; and (4) the WTP of SMEs for flood mitigation activities.

Data collection was carried out through in-depth interviews on SMEs and supported by questionnaires and focus group discussions (FGDs) to gain information on the aspects related to vulnerability to floods and mitigation activities. There were two analytical tools used in the present study, namely flood vulnerability index (Table 1) and economic valuation.

**TABLE 1:** Vulnerability variables and indicators.

Variable	Indicator	Definition
Exposure	Flood frequency	Number of years experiencing extremely high rainfall and severe floods, taken as a proxy
	Flood water depth	Total depth of the floodwater (m)
	Flood duration	Total amount of time the flood persisted in the village (days)
	Elderly	Percentage of workers > 60 years old (%)
	Proximity to river	Total distance of the business location from the river (m)
Sensitivity	Treatment frequency	Number of workers having health problems because of floods
	Access to clean water	The amount of freshwater to be purchased during floods (IDR)
	Income	Total income of SME owners (IDR)
	Migration	Number of workers who resigned and migrated to cities
Adaptive capacity	The condition of river, dikes, sluice gates	River, embankments and sluices condition (%)
	Flood-prone maps	The availability of flood-prone maps (number)
	Education	Percentage of literate workers in the SME (%)
	Distance to health services	Distance travelled to the nearest public health centre (m)
	Evacuation sites	Distance travelled to reach the nearest evacuation site (m)
	Number of NGOs	Total number of NGOs providing relief to the flood victims
	Information access	Total access of SME owners to flood information (number)
	Number of flood camps	The number of flood camps
	Flood awareness	Percentage of workers having assurance (%)
	Emergency services	Number of emergency services
	Early warning of flood	Early flood warning (number)
	Dissemination of flood prevention	The amount of dissemination on flood risk attended by SME owners (number)
	Training of flood prevention	The amount of training on flood risk attended by SME owners (number)

*Source:* Balica et al. (2012), Chaliha (2012), Weis et al. (2016), Neise et al. (2019), Kato & Charoenrat (2018).

Note: Please see the full reference list of the article, Isa, M. & Mardalis, A., 2022, ‘Flood vulnerability and economic valuation of small and medium-sized enterprise owners to enhance sustainability’, *Jàmbá: Journal of Disaster Risk Studies* 14(1), a1306. https://doi.org/10.4102/jamba.v14i1.1306, for more information.

IDR, Indonesian Rupiah; SME, small and medium-sized enterprise.

The flood vulnerability index is determined through compilation of all indices of the aspects of flood vulnerability (Akukwe & Ogbodo 2015; Weis et al. 2016). They consist of exposure, sensitivity and adaptive capacity. Weighting each variable is done by considering the influence of each aspect in shaping the vulnerability aspect. The greater the influence of an aspect, the higher the weight. Weighting is obtained through FGDs with stakeholders related to mitigation activities at the study site. Furthermore, the vulnerability index is determined by multiplying the total scores of all indicators and the weight of exposure, sensitivity and adaptive capacity (Weis et al. 2016). The flood vulnerability index of an area is expressed in the following formula (Isa et al. 2018):


$$Vurnerability=\sum_{i=1}^{3}\left(W_{1}\times X_{1})+(W_{2}\times X_{2})+(W_{3}\times X_{3}\right)$$ [Eqn 1]

Vulnerability = Flood vulnerability index

*W*_1_ = Exposure weight

*X*_1_ = Exposure score

*W*_2_ = Sensitivity weight

*X*_2_ = Sensitivity score

*W*_3_ = Adaptive capacity weight

*X*_4_ = Adaptive capacity score

Economic valuation can be done using the contingency valuation method, namely by determining consumer preferences for the use of natural and environmental resources, as expressed by the WTP in the value of money (Rewitzer et al. 2017). This method is carried out by interviewing respondents about the value and benefits of the natural and environmental resources. It was used to find out the amount of money paid by people to reduce the risks of floods.

### Ethical considerations

This article followed all ethical standards for research without direct contact with human or animal subjects.

## Results and discussion

### Flood vulnerability index

The level of flood-zone vulnerability in Klaten Regency of Central Java, Indonesia, influenced the flood risk. The high risk of flooding hurts the local income and labour absorption and ultimately the regional economic growth (Isa et al. 2015). The high rate of flood risk showed that there were unsolved economic problems related to flood-zone vulnerability and SMEs’ resilience to flood, which depicted inefficiency in flood management. The flood risk portrays the ability of SMEs to cope with flooding. The low flood ratio delineated that SMEs were impervious to flooding.

Adger (2006) and Luers (2005) claimed that sensitivity is an aspect of vulnerability that represents the situation of SME owners in dealing with flooding. It explains the resilience of SME owners. Table 2 shows the index for the indicators of income and frequency of treatment can be classified into high levels. Meanwhile, the index for the indicators of access to clean water and migration can be included in the moderate category. These results imply that the economic and health aspects have the highest effect on sensitivity as the most vulnerable variable to the threat and risks of floods.

**TABLE 2:** Sensitivity index.

Sensitivity indicator	Index
Treatment frequency	0.69
Access to clean water	0.36
Income	0.92
Migration	0.46

Adaptive capacity is the second most vulnerable variable to the threat of floods. Allen (2005), Fussel and Klein (2006) and Isa et al. (2015) argued adaptive capacity as an aspect of vulnerability that explains the ability of a system, including SMEs, to reduce flood risks. Table 3 shows the aspects of evacuation routes that can be classified into the category of high flood vulnerability. It implies the necessity to improve this aspect in order to reduce the risks. Furthermore, several aspects can be included in moderate vulnerability, including the condition of the river, dikes, sluice gates, flood-prone mapping, educational background of an SME’s owner, distance to health services, number of NGOs, number of camps, insurance ownership and number of early warnings. Moreover, the indicators of evacuation sites for the victims, access to information on floods, emergency services, socialisation and trainings are in the category of low vulnerability.

**TABLE 3:** Adaptive capacity index.

Adaptive capacity indicator	Index
The condition of river, dikes, sluice-gates	0.60
Flood-prone mapping	0.48
Educational	0.44
Distance to health services	0.65
Evacuation route	0.83
Evacuation site	0.23
Number of NGOs	0.41
Access to information	0.25
Number of flood camps	0.35
Flood awareness	0.50
Emergency services	0.27
Early warning of the flood	0.50
Dissemination of flood prevention	0.19
Training of flood prevention	0.15

Exposure ranks third as the factor in the vulnerability of non-coastal areas to floods. Weis et al. (2016) asserted that exposure is an aspect that explains the extent to which floods affect SMEs, relating to the vulnerability level, dwelling and flood conditions. Based on Table 4, the index for indicators of flood duration, flood inundation and distance to flood sources (rivers) are in the high vulnerability category. Meanwhile, the frequency of floods and the number of elderly people are included in low vulnerability. Based on this aspect, several factors must get special attention, including the flood duration and flood inundation. Moreover, drainage channels and recharge areas are also vital to eliminate flood inundation and flood duration. In addition, the number of business locations built on the banks of the river must also be reduced. In overall, the key is the enforcement of regional spatial laws and permits in the construction of new buildings or renovations.

**TABLE 4:** Exposure index.

Exposure indicators	Index
Flood frequency	0.29
Flood water depth	0.63
Flood duration	0.42
Elderly	0.27
Distance to flood source	0.60

The flood vulnerability index of an area is estimated by multiplying the total score of all indicators and the weight of exposure, sensitivity and adaptive capacity. The vulnerability index in Klaten was 0.49. This index indicated a medium level of vulnerability. The index is presented in Table 5.

**TABLE 5:** Flood vulnerability index of Klaten Regency.

Exposure		Sensitivity		Adaptive capacity		Vulnerability index
Score	Weight	Score	Weight	Score	Weight	
0.39	0.35	0.68	0.30	0.42	0.35	0.49

Vulnerability index for Exposure = 0.14; Vulnerability index for Sensitivity = 0.20; Vulnerability index for Adaptive capacity = 0.15.

Based on the data and analysis, the vulnerability of flood-prone areas in Klaten regency could be classified in the moderate category, as indicated by the index of 0.34–0.66. The sensitivity aspect has the highest effect on the vulnerability level of an area, followed by adaptive ability and exposure. This finding is different from previous studies conducted in coastal areas, which found that exposure was the most vulnerable aspect to the threat of floods, and it generated numerous risks of floods (Akukwe & Ogbodo 2015; Isa et al. 2018).

Many SMEs are in this region, namely rice mills and producers of furniture, confections, batik and various handicrafts. They are in flood-prone areas, and they have a moderate level of vulnerability. This means that the SMEs are vulnerable to uncertainty regarding flooding, so mitigation is needed to maintain the sustainability of SMEs in this region. Small and medium enterprises will develop well if they are in areas with low vulnerability.

### Flood risks and willingness to pay of flood mitigation

Flood hazards have brought numerous risks to the daily activities of SMEs in the Klaten regency. The risks include damage and losses to buildings and business appliances, trade, agricultural activities, livestock-fisheries and the loss of productivity. One of the adverse impacts for farmers is a decline in production. The business sectors that suffer the most severe damage are livestock and fisheries, buildings and business appliances and agriculture, with the values of Indonesian Rupiah (IDR) 101 020 000 (USD 7064.34), IDR 59 550 000 (USD 4164.34) and IDR 38 700 000, respectively (USD 2706.29). Meanwhile, the fields with the highest loss are livestock and fisheries, agriculture and the loss of productivity, with the values of, respectively, IDR 140 700 000 (9839.16), IDR 57 400 000 (USD 4013.99) and IDR 15 920 000 (USD 1113.29).

The WTP for flood mitigation is an alternative for flood risk reduction from the aspect of individual SMEs’ owners (Kim 2002). Public awareness and willingness to participate in mitigation activities are important to reduce the flood risks. The non-demand curve approach used in the present study is the analysis of WTP. Based on the results of interviews with respondents, there were several classifications of the WTP to flood mitigation on a monthly basis. Approximately 34.87% of respondents were willing to pay for flood mitigation, as much as IDR 100 000 (USD 6.99) to IDR 49 999 (USD 10.49). Meanwhile, 21.71% of respondents were willing to pay for flood mitigation as much as IDR 150 000 (USD 10.49) to IDR 199 999 (USD 13.99); 20.39% of respondents were willing to pay IDR 50 000 (USD 3.50) to IDR 99 999 (USD 6.99); 12.50% of respondents were willing to pay IDR 50 000 (USD 3.50); and 10.53% of respondents were willing to pay more than IDR 200 000 (USD 13.99) (Table 6).

**TABLE 6:** The economic valuation of flood risks in Klaten.

Type of risk	Damage	Loss
Buildings & business appliances	59 550 000.00	-
Trade	8 600 000.00	13 000 000.00
Agriculture	38 700 000.00	57 400 000.00
Livestock and fisheries	101 020 000.00	140 700 000.00
Loss of productivity	-	15 920 000.00

**Total**	**207 870 000.00**	**227 020 000.00**

Note: Values are in Indonesian Rupiah.

Most respondents prefer to use their energy for flood mitigation activities, instead of giving a sum of money. Most of them perceive that flood mitigation should be the government’s responsibility. Therefore, if they are required to contribute in the form of money, they will prefer the minimum amount.

## Conclusion

Based on the flood vulnerability index generated from the perception of SMEs, it can be concluded that Klaten Regency is in the category of moderate flood vulnerability. The sensitive aspect is the most influential aspect of the vulnerability of Klaten regency, followed by the aspects of adaptive ability and exposure.

Furthermore, the economic valuation analysis reveals that the WTP of the SME owners to reduce the flood risks is in the range of IDR 100 000–149 999 (Table 7). As indicated by most of the respondents in the present study, the SME owners perceive that flood mitigation should be carried out by the government, whilst they only serve to support these activities. Therefore, an attempt to raise SME owners’ awareness is required so they will realise their individual role in disaster risk reduction.

**TABLE 7:** The willingness to pay of flood mitigation.

Classification of WTP	Total	Percentage
< 50 000	19	12.50
50 000–99 999	31	20.39
100 000–149 999	53	34.87
150 000–199 999	33	21.71
> 200 000	16	10.53

**Total**	**152**	**100.00**

Note: Values are in Indonesian Rupiah.

WTP, willingness to pay.

Based on the conclusion, several suggestions can be proposed relating to this issue. The suggestions include the following:

1. Collaboration is required between the government, the SME owners and stakeholders related to sustainable flood mitigation, both structural and non-structural.
2. It is suggested for the SME owners to have insurance and improve individual resilience to flood hazards, because these are natural hazards with high risks that can occur anytime. Thus, the SME owners in flood-prone areas have to face a high risk of uncertainty.
3. The National Agency for Disaster Countermeasure (BNPB) should conduct socialisation and trainings to reduce flood risks.
